# Magnitude of effect and sample size justification in trials supporting anti-cancer drug approval by the US Food and Drug Administration

**DOI:** 10.1038/s41598-023-50694-0

**Published:** 2024-01-03

**Authors:** Michelle B. Nadler, Brooke E. Wilson, Alexandra Desnoyers, Consolacion Molto Valiente, Ramy R. Saleh, Eitan Amir

**Affiliations:** 1 Division of Medical Oncology and Hematology, Princess Margaret Cancer Centre and Department of Medicine, The University of Toronto, Toronto, ON Canada; 2 Kingston Health Sciences Centre, Kingston, ON Canada; 3 Université de Sherbrooke, Sherbrooke, QC Canada; 4 Division of Medical Division of Medical Oncology, McGill University Health Centre, Montreal, QC Canada

**Keywords:** Cancer, Cancer, Statistical methods

## Abstract

Approval of drugs is based on randomized trials observing statistically significant superiority of an experimental agent over a standard. Statistical significance results from a combination of effect size and sampling, with larger effect size more likely to translate to population effectiveness. We assess sample size justification in trials supporting cancer drug approvals. We identified US FDA anti-cancer drug approvals for solid tumors from 2015 to 2019. We extracted data on study characteristics, statistical plan, accrual, and outcomes. Observed power (*P*_*obs*_) was calculated based on completed study characteristics and observed hazard ratio (HR_*obs*_). Studies were considered over-sampled if *P*_*obs*_ > expected with HR_*obs*_ similar or worse than expected or if *P*_*obs*_ was similar to expected with HR_*obs*_ worse than expected. We explored associations with over-sampling using logistic regression. Of 75 drug approvals (reporting 94 endpoints), 21% (20/94) were over-sampled. Over-sampling was associated with immunotherapy (OR: 5.5; *p* = 0.04) and associated quantitatively but not statistically with targeted therapy (OR: 3.0), open-label trials (OR: 2.5), and melanoma (OR: 4.6) and lung cancer (OR: 2.17) relative to breast cancer. Most cancer drug approvals are supported by trials with justified sample sizes. Approximately 1 in 5 endpoints are over-sampled; benefit observed may not translate to clinically meaningful real-world outcomes.

## Introduction

Decisions on regulatory approval of drugs are based typically on randomized trials observing statistically significant superiority of an experimental agent over an established standard. Recently, the American Statistical Association has highlighted the limitations of basing decisions on *p*-values emphasizing that statistical significance can be the result of large effect size, high statistical power, or a combination of the two^1,2^.

Randomized trials supporting drug approval have restrictive eligibility criteria which sub-optimally represent patients treated in routine clinical practice^3,4^. This can lead to differences in outcomes between patients treated in trials and those treated in the real-world setting^5–7^. Compared to clinical trials, some treatments delivered in the clinical setting result in less beneficial effect and greater toxicity^8–10^. This scenario is referred to as the efficacy-effectiveness gap^11^.

While regulatory approval is based predominantly on the observation of statistically significant results from adequately controlled studies, statistical significance does not always translate to clinical meaningfulness. Prior work on clinically meaningful benefit has defined this as a noticeable and/or valuable effect experienced by the patient^12^. Clinically meaningful change has been defined for OS as a hazard ratio (HR) of 0.8 or lower; for intermediate endpoints, higher magnitudes of effect have been suggested^13^. Assuming justified sample size^14^, a clinical trial with an endpoint that is statistically significant due to a larger than expected effect size is more likely to translate to improved outcomes in practice^15^. Conversely, an endpoint which maintains statistical significance despite an effect size that is lower than expected may be due to over-sampling and is less likely to translate to improved real-world outcomes.

Over-sampling has been defined previously as intentionally sampling of typically under-represented groups to make up a larger proportion of a survey sample than they do in the population^16^. This can improve external validity. Conversely, oncology drug trials have more restrictive eligibility criteria, so a smaller effect size may result in less clinically meaningful benefit in practice for the average patient^17^. It is unknown if trials supporting approval of anti-cancer drugs are statistically significant due to a large magnitude of effect or over-sampling.

In this article, we assess clinical trial endpoints supporting recent cancer drug approvals, explore justification for sample sizes, and estimate the proportion in which statistical significance may have resulted from over-sampling. We hypothesized that most endpoints would have higher power than planned due to over-sampling, rather than due to increased magnitude of effect.

## Methods

### Data source and eligibility

We searched the US Food and Drug Administration (FDA) drug approvals website^18^ to identify drug approvals for solid tumors (excluding lymphomas) from January 1, 2015 to December 31, 2019. We excluded hematologic malignancies, as is the standard for oncology studies, due to differences in treatment goals and in commonly used trial endpoints. There were no restrictions to type of anti-neoplastic agent. This study was exempt from institutional review board approval since it comprised exclusively of the use of publicly available data.

We included prospective, randomized trials (of any phase) with a primary outcome of disease or recurrence-free survival, progression-free survival (PFS), metastasis-free survival, or overall survival (OS). Eligible studies needed to include data detailing the statistical plan (in the manuscript or supplementary appendices), including the targeted/expected effect size (referred to as expected henceforth), accrual time, duration of follow-up, type I error (alpha) and expected power. Corresponding authors were contacted when data were not available. Studies were excluded if they were non-inferiority trials or if FDA approval was withdrawn since the initial approval.

### Data extraction

One author (MBN) retrieved the relevant manuscripts and supplementary appendices of the report of trials supporting each drug approval. Data extraction and calculations were performed by two authors (MBN and BEW). Discrepancies were resolved by consensus and/or with the involvement of a third author (EA). The following data were extracted for the intent-to-treat analysis for each study endpoint: type of malignancy, drug type, primary outcome(s) and secondary outcome (if it was OS), blinding versus open-label, alpha, number of patients in the experimental arm, number of patients who withdrew consent or were lost-to follow-up, expected HR in the statistical plan, observed HR, median duration of time-to-event in the control arm (for outcome of interest), accrual start and end dates, data cut-off date, ratio of control to experimental group, and expected power defined by the study’s statistical plan.

Drug types were categorized as chemotherapeutic agents, hormonal therapy, immunotherapy, other monoclonal antibodies, PARP-inhibitors, and targeted small molecules. Immunotherapy was grouped separately (despite it being a monoclonal antibody) because it has a unique mechanism of action, eliciting the host’s immune response rather than an oncogenic target as is the case with most other monoclonal antibodies. Similarly, we grouped PARP-inhibitors separately given their target is typically a germline rather than a somatic alteration. This unique mechanism of action, multiple drugs in class and overall good tolerability in contrast to other small molecules used in oncology warrant assessment in a single subgroup. The expected HR* (HR*_*exp*_*)* and expected power* (P*_*exp*_*)* was also extracted for each endpoint. A separate author (CMV) extracted and calculated the American Society of Clinical Oncology Value Framework (ASCO-VF) version 2 scores. The ASCO-VF is a tool designed to identify drugs of substantial value considering both efficacy and safety/tolerability with scores of 45 or more defined as clinical value^19,20^. Scores were calculated with and without correction for toxicity, safety, or quality of life.

The total accrual time (in months) was calculated as {(accrual end – accrual start) / 30.4375} and follow-up time after end of recruitment “F” (in months) was calculated as {(data cut off time – accrual end month) / 30.4375}. Both were rounded to the nearest half-integer. If data cut-off was not available, it was calculated by taking the mid-point of accrual time and adding the reported median follow-up. If the median number of months of the outcome of interest was not available, it was calculated using the following formula: *t* log_e_(1/2)/log_e_(*p*) where *p* is the probability that a control subject survives until time *t*. Additional methods and assumptions are reported in Supplementary Table 1.

### Data synthesis and statistical analysis

In order to explore justification for sample size and potential for over-sampling, first, we estimated the observed power (*P*_*obs*_) of each endpoint. This was done by inputting the following variables into the *Power and Sample Size* calculator (version 3.0, January 2009)^21^: number of patients in experimental arm, *HR*_*exp*_, observed HR *(HR*_*obs*_), median duration of time-to-event in the control arm (for outcome of interest), accrual start and end dates, data cut-off date, ratio of control to experimental group, and *P*_*exp*_. *P*_*obs*_ was calculated for each trial’s primary endpoint (and secondary endpoint if it was OS). The absolute difference between observed and expected power was calculated (ΔP_O-E_ = *P*_*obs*_−*P*_*exp*_).

#### Definitions

By convention, we defined equivalent power using a 5% spread (i.e. *P*_*exp*_ was considered similar to *P*_*obs*_ if it was within ± 2.5%) and under-powered endpoints as ΔP_O-E_ < 2.5%. Similarly, *HR*_*obs*_ was considered similar to *HR*_*exp*_ if the absolute difference between the two was within 0.025. We defined study endpoints as *over-sampled* if a) P_*obs*_ was larger than P_exp_ and *HR*_*obs*_ had a similar or worse magnitude of effect than *HR*_*exp*_ or b) if the endpoint was similarly powered but *HR*_*obs*_ was worse than *HR*_*exp*_.

In order to explore the validity of our definition of oversampling, we performed a post-hoc analysis exploring the association between this definition and ASCO-VF scores. We used the tool initially in an unadjusted manner and subsequently without correction for toxicity, safety, or quality of life.

#### Sensitivity analyses

Given there is no definition for over-sampling in the literature, a series of post-hoc sensitivity analyses were performed. This included defining equivalent power using a 10% spread (i.e. *P*_*exp*_ considered similar to *P*_*obs*_ if within a difference of ± 5%) and equivalence between *HR*_*obs*_ and *HR*_*exp*_ if the absolute difference was within 0.01 or 0.05. Additional post-hoc sensitivity analyses included excluding studies where follow-up time after end of accrual was 0, was estimated (resulting in a value of zero or greater than zero), both together, and excluding endpoints where median outcome of interest was calculated rather than extracted. Finally, we performed a sensitivity analysis using only one end-point per trial to avoid colinear data. We utilized a hierarchy preferring primary to secondary endpoints and in trials with co-primary endpoints selecting OS over intermediate endpoints.

Associations between any over-sampled endpoint and study characteristics were explored using logistic regression. The regression was repeated for any sensitivity analysis where the proportion of over-sampled trials differed from the primary analysis by more than 5% and using only one endpoint per trial. Statistical significance was defined as *p* < 0.05. No corrections were applied for multiple significance testing. The Burnand criteria for quantitative significance^22^ were used to evaluate the magnitude of effect of associations irrespective of statistical significance in the context of low power.

## Results

**Figure 1:**
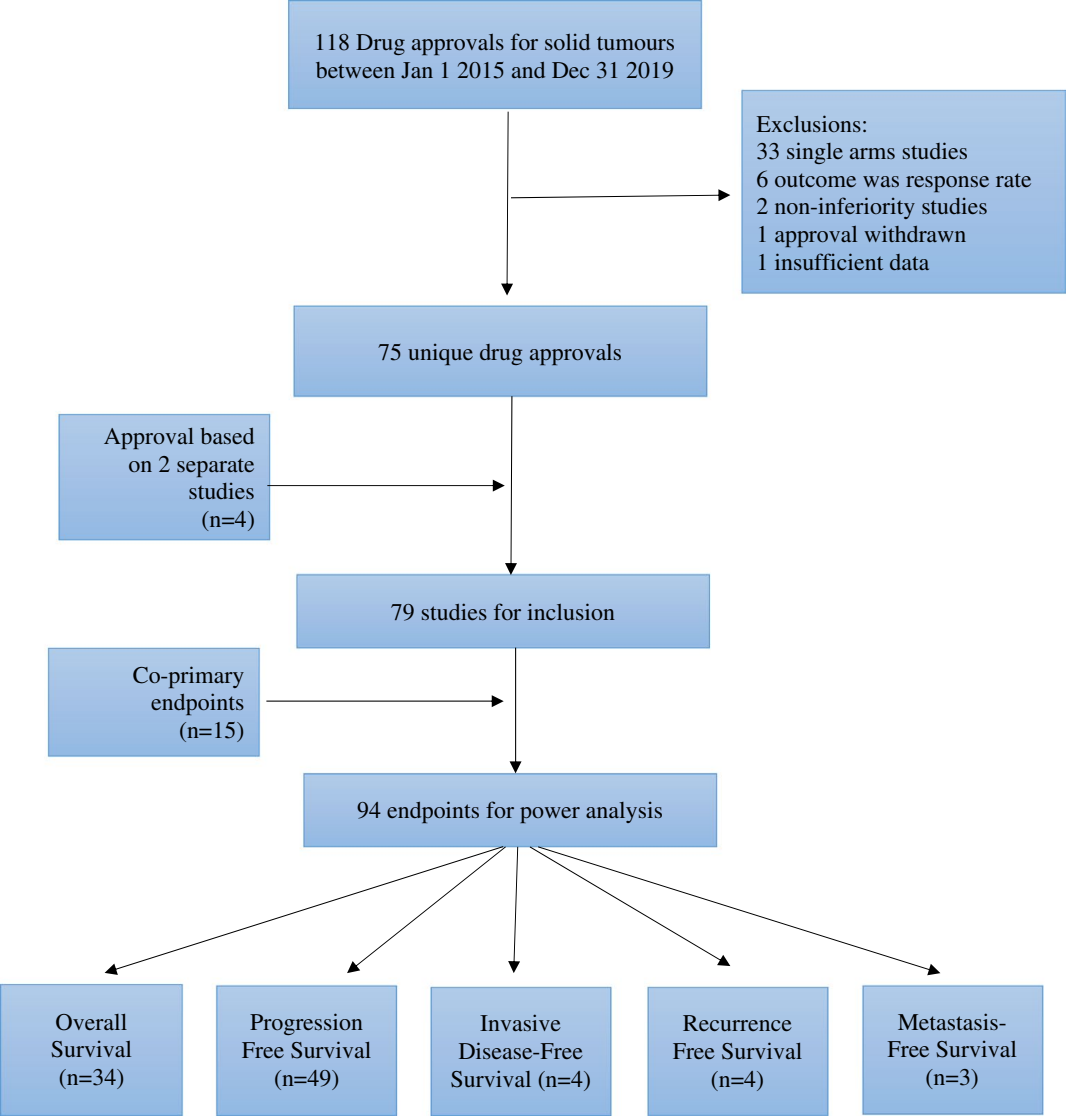
Trial Selection.

The search identified 118 unique drug approvals, of which 75 (70 phase 3; 5 phase 2) met our inclusion criteria (Fig. 1). Reasons for exclusions were single arm studies, drug taken off the market due to lack of efficacy in a post-marketing trial (olaratumab for soft-tissue sarcoma), and data unavailable despite contact with study authors (olaparib maintenance in relapsed *BRCA1/2*-mutated ovarian cancer). Among the 75 included drug approvals, 4 were based on two separate manuscripts, and 15 had a co-primary endpoint (or a secondary endpoint of OS). Consequently, the analysis cohort comprised a total of 94 trial endpoints for which observed power could be calculated. An overview of trial (n = 75) and endpoint (n = 94) characteristics is found in Table 1.

**Table 1:** Characteristics of FDA Drug Approvals between 2015 and 2019.

Approval characteristic	Number (%)
	(n = 75)
Study phase	
Phase 2	5 (6.7)
Phase 3	70 (93.3)
Year of drug approval	
2015	15 (20)
2016	8 (10.7)
2017	16 (21.3)
2018	19 (25.3)
2019	17 (22.7)
Type of cancer	
Lung	18 (24)
Breast	14 (18.7)
Melanoma	9 (12)
Renal cell carcinoma	8 (10.7)
Prostate	6 (8)
Ovary/primary peritoneal	5 (6.7)
Hepatocellular carcinoma	3 (4)
Upper GI and pancreas	3 (4)
Colorectal	2 (2.7)
Sarcoma	2 (2.7)
Head and neck	2 (2.7)
Thyroid	1 (1.3)
Neuroendocrine	1 (1.3)
Bladder	1 (1.3)
Type of drug	
Targeted small molecules	27 (36.0)
Immunotherapy	23 (30.7)
Hormonal therapy agents	6 (8.0)
PARP-inhibitors	6 (8.0)
Monoclonal antibodies	5 (6.7)
Chemotherapeutic agents	5 (6.6)
Combination therapy*	3 (4.0)

Endpoint characteristic	Mean ± SD (range)
	(n = 94)
Alpha	0.049 ± 0.05
	(0.001–0.4)
Expected power	87.4% ± 7.58
	(40–99%)
Observed power	91.6 ± 0.16
	(18.5–100%)

*Immunotherapy plus small molecule or antibody–drug conjugate.

For 11 endpoints (10 trials), follow-up time after end of accrual (“F”) was either estimated or was ≤ 0 by design. For 5 endpoints (10 trials), data-cut off was estimated and resulted in F > 0. For 4 endpoints (3 trials), reported data cut-off was either before or on the date of end-accrual. One trial did not provide data-cut-off or a median follow-up time and in one trial the estimated follow-up time resulted in data cut-off occurring before end of accrual (presumably due to non-linear accrual). For all these trials, F was defined as zero. The median endpoint time for the outcome of interest in the control group was not reported for 9 trials (10 end points ) as the median was not reached.

Among the 94 analyzable endpoints, 3 trial endpoints (3%) were well-powered, 19 (20%) were under-powered, and 72 (77%) had P_*obs*_ larger that P_exp_. Statistical metrics of these study endpoints studies are shown in Table 2. A histogram of ΔP_O-E_ is provided in Supplementary Fig. 1 and of difference in HR in Supplementary Fig. 2. In the sensitivity analysis using the 10% spread, 19 (20%) endpoints were categorized as well-powered, 17 (18%) under-powered, and 58 (62%) had P_*obs*_ larger that P_exp_.

**Table 2:** Metrics for Evaluable Study Endpoints.

	P_*obs*_ larger that P_exp_ (n = 72)	Under-powered Endpoints (n = 19)	Well-powered Endpoints (± 2.5%) (n = 3)
Expected power (%)	87.3 ± 7.75	86.4 ± 6.6	96 ± 4.3
Observed power (%)	98.2 ± 3.5	65.6 ± 19.7	96.5 ± 5
ΔP_O-E_ n (%)			
< − 2.5%		19 (20%)	
− 2.5 to + 2.5%			3 (3%)
2.51–5%	14 (15%)		
5.1–10%	39 (42%)		
10.1–20%	16 (17%)		
> 20%	3 (3%)		
Expected HR (n = 91)*	0.667 ± 0.07 (n = 69)	0.69 ± 0.08	0.66 ± 0.06
Observed HR	0.54 ± 0.13	0.76 ± 0.6	0.71 ± 0.09
Alpha	0.053 ± 0.06	0.038 ± 0.019	0.019 ± 0.026
Sample size – experimental arm	362.8 ± 225	414.7 ± 497.5	475 ± 145
Median outcome time in control arm (months)	14.8 ± 24.6	30.9 ± 79.4	10 ± 2.8

*3 studies the expected HR was not available and therefore observed power could not be calculated.

In 3 endpoints, the statistical plan did not provide *HR*_*exp*_, therefore assessment of over-sampling was based on 91 endpoints (Table 3). Of all trial endpoints, 19 (21%) were considered over-sampled. Among evaluable endpoints with P_*obs*_ larger that P_exp_ (n = 69), 17 (25%) were over-sampled. Results of sensitivity analyses are shown in supplementary table 2A–F. Between 16 and 29% of end-points were over-sampled across six analyses resulting in an average of 20% over-sampled end-points. Results of sensitivity analyses excluding end-points where data points were estimated were unchanged (supplementary table 3A–D). In the sensitivity analysis with one end-point per trial, 18% of end-points are over-sampled (supplementary table 4).

**Table 3:** Observed Power and Assessment of Over-sampling (n = 91).

	{P_obs_ > (P_exp_ + 2.5%)} (n = 69)	Well-powered (n = 3)	Under-powered {P_obs_ < (P_exp_—2.5%)} (n = 19)
*HR*_*obs*_ better magnitude of effect than *HR*_*exp*_ (n = 54)	52	1	1
*HR*_*obs*_ similar magnitude of effect as *HR*_*exp*_ (n = 15)	**12**	0	3
*HR*_*obs*_ worse magnitude of effect than *HR*_*exp*_ (n = 22)	**5**	**2**	15

Over-sampled endpoints are highlighted in bold. Study endpoints were considered to be over-sampled if the endpoint had P_*obs*_ larger that P_exp_ and *HR*_*obs*_ similar or worse magnitude of effect than *HR*_*exp*_ OR if the endpoint was well-powered and *HR*_*obs*_ worse magnitude of effect than *HR*_*exp.*_ Nineteen endpoints were considered oversampled (19/91, 20%).

In the unadjusted analyses, there was no difference in ASCO-VF scores between trials defined as oversampled and those that were not (mean 44.4 vs. 45.8, *p* = 0.40). However, when ASCO-VF was not adjusted for safety/tolerability, there appeared to be a modest difference in scores which approached, but did not meet statistical significance (mean 43.1 versus 47.9, *p* = 0.13). This suggests that trials defined as oversampled may be less likely to meet thresholds for substantial clinical value.

Over-sampling was both statistically and quantitatively associated with immunotherapy (OR: 5.5, *p* = 0.04) while quantitative, but not statistical associations were observed for targeted therapy relative to other types of therapy (OR: 3.0, *p* = 0.2), open-label trials compared to double-blind trials (OR: 2.5, *p* = 0.08), and melanoma (OR: 4.6, *p* = 0.11) and lung (OR: 2.17, *p* = 0.39) cancers relative to breast cancer. There were no associations with year of approval, type of endpoint, or the number of patients lost to follow-up or who withdrew consent (Table 4). The repeated regressions for the sensitivity analyses are shown in Supplementary Tables 5A–C. For analyses in which fewer studies were categorized as over-sampled, quantitative significance was attenuated modestly but retained similar quantitative associations and the association with immunotherapy lost statistical significance. In the sensitivity analysis with more end-points categorized as over-sampled, the associations with open-label trials (OR: 3.22, *p* = 0.02) and melanoma relative to breast cancer (OR: 9.1, *p* = 0.02) became statistically significant.

**Table 4:** Sampling characteristics of Over- and Under-sampled Endpoints (n = 91).

	Over-sampled studies (n = 19)	Not over-sampled (n = 72)	OR	*P*
Expected power % (mean ± SD)	88.6 ± 5.6	86.9 ± 8.1	1.03 (0.96–1.13)	0.37
Alpha % (mean ± SD)	4.6 ± 5.1	5.1 ± 5.7	0.98 (0.88–1.09)	0.73
Sample size – exp arm (mean ± SD)	347.6 ± 300.5	384.6 ± 301	0.99 (0.997–1.001)	0.63
m1 control (months)	18.3 ± 41.8	18.1 ± 42.6	1.00 (0.98–1.01)	0.99
Study year			0.86 (0.61–1.21)	0.40*
2015, n (%)	7 (36.8%)	12 (16.7%)		
2016, n (%)	1 (5.3%)	9 (12.5%)		
2017, n (%)	4 (21.1%)	13 (18.1%)		
2018, n (%)	0	24 (33.3%)		
2019, n (%)	7 (36.8%)	14 (19.4%)		
Type of therapy*				
Other, n (%)	2 (10.5%)	24 (33.3%)	1	
Targeted therapy, n (%)	6 (31.6%)	24 (33.3%)	3 (0.55–16.4)	0.2
Immunotherapy, n (%)	11 (57.9%)	24 (33.3%)	5.5 (1.1–27.4)	0.04
Type of endpoint				
Other, n (%)	10 (52.6%)	48 (66.7%)	1	
OS, n (%)	9 (47.4%)	24 (33.3%)	1.82 (0.65—5.0)	0.26
Disease site				
Breast, n (%)	2 (10.5%)	13 (18.1%)	1	
Lung, n (%)	6 (31.6%)	18 (25%)	2.17 (0.37–12.5)	0.39
Melanoma, n (%)	5 (26.3%)	7 (9.7%)	4.6 (0.71–30.4)	0.11
Other, n (%)	6 (31.6%)	34 (47.2%)	1.14 (0.2–6.42)	0.88
Blinding				
Double blind, n (%)	7 (36.8%)	43 (59.7%)	1	
Open label, n (%)	12 (63.2%)	29 (40.3%)	2.56 (0.89–7.14)	0.08
Loss to follow-up or withdrawal (mean ± SD)		(n = 70)		
	24.05 ± 16.93	24.9 ± 33.9	0.99 (0.98–1.01)	0.91

*P for trend.

## Discussion

In this study, we explored whether sample size calculations of trials supporting cancer drug approval were justified. Results showed that for most drug approvals in solid tumors, statistical significance of the primary endpoint resulted primarily due to better than anticipated effect size. This is a reassuring result as it is likely that in the setting of statistical significance and large effect size, efficacy observed in clinical trials may translate to effectiveness in the real-world setting. Clinicians can be assured that many of the oncologic treatments studied in these trials will benefit their patients. A drug with robust efficacy should maintain an effect size and statistical significance even in the face of clinical trial participants who are more heterogeneous. This is relevant to future trial design as clinicians, researchers, and trialists may feel confident decreasing barriers to trial entry; this would improve trial access and enrollment for more diverse populations and also allow for more generalizable trial data^23^.

Another promising finding is that sufficient data were reported in the included studies to allow reproduction of sample size calculation for all but 3 endpoints. This suggests that the quality of reporting and justification of sample size is consistent with CONSORT guidelines^24^ and has improved for the recent oncology trials reported in this study compared to a report from 2015 suggesting that only 28% of trials provided all of the required parameters for a sample size calculation^25^.

Importantly, in approximately 20% of all endpoints supporting cancer drug approval, there was an effect size similar or of lesser magnitude than expected. Statistically significant results of these studies are likely due to over-sampling. This could occur directly by recruitment of more patients than required to show statistical significance or (intentionally or unintentionally) manipulating other variables in the sample size calculation, such as extending the follow-up time or increasing alpha or beta (as described below). This suggests that sample size calculations in these studies were not justified. This finding deserves attention as it could impede the translation of clinical trial results to the real world. In these circumstances, the benefit-risk ratio of certain drugs may become unfavourable^9,26^.

While we could not evaluate the reason for over-sampling, we did observe that retention of high observed statistical power despite smaller than anticipated effect size was associated with immunotherapy, targeted therapy, melanoma, lung cancer and was more common in open-label studies. The association with targeted therapy is concerning as these drugs have been associated with a high prevalence of grade 3 toxicity in registration trials^27,28^ and often require dose adjustments in response to toxicity especially in the real-world setting^29^. Drugs studied in open label trials have been shown to provide a lower magnitude of benefit than those evaluated in blinded studies^30^. Taken together, the combination of over-sampling, lower magnitude of effect and higher toxicity is concerning as this may also impact negatively on the efficacy-effectiveness gap.

When planning and conducting a trial, oversampling may occur unintentionally and/or may have adequate justification. Prediction of expected outcomes and rate of events in clinical trials is challenging especially if there are few informative data from earlier phase trials. While it has been suggested previously that stronger evidence of biologic effect should be required before a new drug enters phase III testing^31^, this can result in delays to getting a drug to market. Due to the cost, resources, and time taken to run a clinical trial, clinical trialists likely prioritize preventing a type 2 error (under-powering) than type 1 error (albeit typically set conventionally). This can result in the observed findings of over-sampling described in this article. Opportunities that could mitigate the consequences of over-sampling include reporting of observed power in trial reports to allow all stakeholders to decide whether observed benefit is meaningful irrespective of statistical significance. Additionally, regulators could approve drugs supported by over-sampled trials with the condition that post-marketing real-world studies confirm the benefit observed in the registration trial. The results of such post-marketing studies could also provide a better estimate of effectiveness and toxicity both for clinical decision-making and for informing health technology assessments^9^.

The power of a trial describes the avoidance of a false negative result. By convention, investigators and statisticians consider a trial to be adequately powered if it has at least an 80% chance of detecting a significant effect when it truly exists. It is important to note that this value is arbitrary. In our study, we investigated observed power relative to the power defined by the statistical plan, which could have been set below, at, or above 80%. The numerical value of the power is an important consideration when judging whether trial results are clinically meaningful or not and should be justified^32,33^. For example, if a cheap and simple intervention provides benefit, one could justify an increase in power of a planned study^34^. For a treatment with substantial cost or unfavorable safety and tolerability metrics it may not desirable to power a trial in order to identify a small magnitude of effect^35^.

Although it can be justifiable not to follow convention, we report a few observations which deviate considerably from usual standards. One trial endpoint had a *P*_*exp*_ of 40%, although this was a secondary endpoint^36^. In another, *P*_*exp*_ changed from 90 to 95% after initiation of accrual without a clear explanation^37^. Of all end-points, 14% had a *P*_*exp*_ of 95% or greater. These endpoints may or may not have met our definitions for over-sampling but setting power at this level will result in some over-sampling. Similarly, 4 endpoints^38–41^ had an alpha > 0.05 (0.2, 0.24, 0.3, and 0.4). There was no clear justification for this, although all studies were phase 2 and/or in rare disease sites. Finally, some drugs were approved for sub-groups which were not part of the study’s statistical plan (for example, drug approved regardless of a marker status, but the statistical plan powered for the biomarker-specified subgroup). Greater transparency about the data supporting these statistical plans would be welcome.

This study has limitations. First, there is no established definition of over-sampling, so we determined a definition based on prior literature and available data. We explored the validity of our definition by exploring associations with the ASCO-VF. Several sensitivity analyses confirmed our estimated was accurate; however, given the novelty of this estimate, there is no way to assess how it compares to non-oncology trials. Similarly, the concept of “observed power” is debated in the literature, with some suggesting this is a function of *p*-value. We chose to use this as we required a measure that could compare observed results to the original statistical plan. Second, we assessed trials which were randomized, superiority trials. Some cancer drugs are approved on the basis of single arm studies or subgroup analyses^30^. While it is possible to calculate observed power for single arm studies, this power is related to precision of measurement rather than comparative efficacy. This is a different outcome than the objective of this study which focused on comparative time-to-event outcomes. Third, some of our definitions of equivalent power and effect size were arbitrary. However, sensitivity analyses did not suggest that this impacted on estimates of over-sampling or associations therewith. Fourth, we could not determine the specific causes of over-sampling, and there could have been reasons beyond the control of the trialists for this. Fifth, we were limited in evaluating associations with over-sampling due to the heterogeneous nature of the dataset, low power, and potential for autocorrelation. It is important to specifically note that there were insufficient studies to be able to fit a multivariable model adequately and therefore the primary analysis violates the assumption of independent variables. Autocorrelation could have occurred with two endpoints from a similar trial and/or other variables (such as immunotherapy use correlating with year and disease site). Despite these limitations, we showed that approximately 1 in 5 endpoints leading to FDA approvals of cancer drugs are over-sampled, which could limit real-world effectiveness.

In conclusion, most cancer drug approvals have robust sample size justification and are supported by studies in which statistical significance is driven by a greater than anticipated effect size. This is an encouraging result for both clinicians and patients. Approximately 1 in 5 endpoints supporting drug approval are likely over-sampled. In this setting, benefit observed in RCTs may not translate to the real-world setting. Real-world effectiveness studies should be prioritized for these scenarios.

### Supplementary Information

Supplementary Table 2.

Supplementary Information.

## Data Availability

The food and drug administration (FDA) has a public database for all drug approvals. This study used the list of specific oncology (cancer) / hematologic malignancies approval notifications available from this website: https://www.fda.gov/drugs/resources-information-approved-drugs/oncology-cancer-hematologic-malignancies-approval-notifications.
