# Supplementary material for: Magnitude of effect and sample size justification in trials supporting anti-cancer drug approval by the US Food and Drug Administration
Source: Sci Rep. 2024 Jan 3;14:459. doi: 10.1038/s41598-023-50694-0 (PMC10764749; doi:10.1038/s41598-023-50694-0)
Supplement: Supplementary file 1 — Supplementary Table 2. [file 41598_2023_50694_MOESM1_ESM.docx]

Supplementary Table 2: Unique drugs, Manufacturer, year and indication of FDA approval

| **Drug Name** | **Manufacturer** | **Year of FDA Approval** |
| --- | --- | --- |
| Abemaciclib | Eli Lilly | 2017 - metastatic hormone-receptor positive, HER2 negative breast cancer with disease progression after endocrine therapy  2018 – 1^st^ line for metastatic hormone-receptor positive, HER2 negative breast cancer with disease progression after endocrine therapy |
| Abiraterone | Janssen Biotech | 2018 – 1^st^ line metastatic high-risk castrate-sensitive prostate cancer in combination with prednisone |
| Afatinib | Boehringer Ingelheim | 2018 – 1^st^ line for metastatic NSCLC with non-resistant EGFR mutations |
| Alectinib | Roche | 2015 – metastatic NSCLC with ALK mutation, after crizotinib  2017 – 1^st^ line for metastatic NSCLC with ALK mutation |
| Alpelisib | Novartis | 2019 – 2^nd^ line metastatic hormone-receptor positive, HER2 negative PIK3CA mutated breast cancer in combination with fulvestrant after endocrine based regimen |
| Apalutamide | Janssen Biotech, Inc | 2018 – non-metastatic castrate-resistant prostate cancer  2019 – metastatic castrate-sensitive prostate cancer  mCSPC |
| Atezolizumab | Roche | 2016 – metastatic urothelial carcinoma after platinum-based therapy  2016 – metastatic NSCLC after platinum-based therapy  2018 – metastatic urothelial cancer not eligible for cisplatin therapy (in combination with pembrolizumab)  2018 – metastatic non-squamous NSCLC (no EGFR mutation) in combination with paclitaxel, carboplatin, and bevacizumab  2019 – metastatic triple negative breast cancer, PD-L1 ≥1%  2019 – 1^st^ line treatment for extensive-stage small cell lung cancer with carboplatin and etoposide  2019 – 1^st^ line for metastatic NSCLC with no EGFR/ALK mutation in combination with nab-paclitaxel and carboplatin |
| Axitinib | Pfizer | 2019 – 1^st^ line metastatic RCC, in combination with pembrolizumab  2019 – 1^st^ line metastatic RCC in combination with avelumab |
| Avelumab | Merck | 2017 – metastatic merkel cell cancer  2017 – metastatic urothelial cancer after platinum based therapy |
| Bevacizumab | Roche | 2018 – stage 3 or 4 epithelial ovarian, fallopian tube, or primary peritoneal cancer in combination with carboplatin & paclitaxel followed by single agent bevacizumab. |
| Brigatinib | Ariad Pharmaceuticals | 2017 – metastatic ALK-positive NSCLC after crizotinib |
| Cabazitaxel | Sanofi | 2017 – metastatic castrate-resistant prostate cancer after taxane-based therapy |
| Cabozantinib | Exelixis | 2016 – metastatic RCC after anti-angiogenic therapy  2017 – 1^st^ line for metastatic RCC  2019 – metastatic HCC previously treated with sorafenib |
| Cemiplimab | Regeneron Pharmaceuticals | 2018 – metastatic cutaneous squamous cell carcinoma who are not candidates for curative surgery or radiation |
| Ceritinib | Novartis | 2017 – metastatic NSCLC, ALK-positive |
| Cobimetinib | Genetech | 2015 – BRAF V600E/K mutated metastatic melanoma with vemurafenib |
| Crizotinib | Pfizer | 2016 – ROS1-positive metastatic NSCLC |
| Dabrafenib & Trametinib | Novartis | 2015 – metastatic melanoma after platinum-based therapy  2017 – metastatic NSCLC with BRAF V600E/K mutation  2018 – adjuvant treatment for melanoma with BRAF V600E/K mutation  2018 – metastatic anaplastic thyroid cancer with BRAF V600E/K mutation |
| Dacomitinib | Pfizer | 2018 – 1^st^ line metastatic NSCLC with EGFR exon 19 or L858R mutation |
| Darolutamide | Orion Corporation & Bayer | 2019 – non-metastatic castrate resistant prostate cancer |
| Durvalumab | Medimmune / AstraZeneca | 2017 – metastatic urothelial cancer after platinum-based therapy  2018 – Stage 3 NSCLC after concurrent platinum-based chemotherapy and radiation (without progression) |
| Encorafenib and Binimetinib | Array, BioPharma Inc. | 2018 - metastatic melanoma with V600E/K mutation |
| Enfortumab Vedotin | Seagan (now part of Pfizer) | 2019 – metastatic urothelial cancer with prior receipt of platinum and immunotherapy |
| Entrectinib | Roche | 2019 – metastatic / unresectable solid tumors with neurotorphic tyroside receptor kinase (NTRK) gene fusion with no satisfactory standard therapy |
| Enzalutamide | Medivation (now part of Pfizer) | 2018 – non-metastatic castrate-resistant prostate cancer  2019 – metastatic castrate-sensitive prostate cancer |
| Erdatifinib | Astex | 2019 - metastatic urothelial cancer with FGFR 2 or 3 genetic alterations |
| Eribulin | Eisai & Merck | 2016 - metastatic liposarcoma after anthracycline |
| Everolimus | Novartis | 2016 – metastatic, well-differentiated non-functional, neuroendocrine tumors of gastrointestinal or lung origin |
| Gefitinib | AstraZeneca | 2015 – metastatic NSCLC with EGFR mutation |
| Iobenguane I 131 | Progenics | 2018 – metastatic pheochromocytoma or paraganglioma (rare adrenal glad tumours) that are iobeguane scan-positive |
| Ipilimumab | Bristol Myers Squibb | 2015 – melanoma, adjuvant therapy  2018 – MSI-high or dMMR metastatic CRC following fluoropyrimidine, oxaliplatin, and irinotecan |
| Irinotecan liposome | Ipsen Biopharm | 2015 – metastatic pancreatic cancer (post progression with gemcitabine, in combination with fluorouracil based therapy) |
| Lenvatinib | Eisai | 2015 – locally recurrent/metastatic progressive thyroid cancer  2016 – Metastatic RCC after anti-angiogenic therapy in combination with everolimus  2018 – 1^st^ line treatment for metastatic HCC  2019 – metastatic endometrial cancer (not MSI-high or dMMR) after prior therapy (with pembrolizumab) |
| Lorlatinib | Pfizer | 2018 – ALK positive metastatic NSCLC after crizotinib and at least one other ALK inhibitor |
| Necitumumab | Eli Lilly | 2015 – metastatic NSCLC, first line in combination with gemcitabine and cisplatin |
| Neratinib | Puma Biotechnology (Pfizer) | 2017 – extended adjuvant therapy for early stage HER2 positive breast cancer, after trastuzumab |
| Niraparib | GlaxoSmithKline | 2017 – recurrent epithelial ovarian, fallopian tube, or pirmary peritoneal cancer, who are in CR or PR to platinum-based therapy  2019 – advanced ovarian, fallopian, or primary peritoneal cancer treated with 3 or more chemotherapy regimens with HRD positive status with disease progression > 6 months after response to last platinum |
| Nivolumab | Bristol Myers Squibb | 2015 – metastatic squamous NSCLC after platinum  2015 – metastatic adenocarcinoma NSCLC after platinum  2015 – BRAF wild type metastatic melanoma, in combination with ipilimumab  2015 – metastatic RCC, post anti-angiogenic therapy  2016 – metastatic squamous cell carcinoma of head and neck after platinum-based therapy  2017 – metastatic urothelial cancer after platinum-based therapy  2017 – dMMR and MSI-high metastatic CRC after fluoropyrimidine, oxaliplatin, and irinotecan  2017 – metastatic HCC after sorafenib  2017 – Adjuvant treatment for melanoma  2018 – 1^st^ line intermediate or poor risk metastatic RCC in combination with ipilimumab  2018 – metastatic small-cell lung cancer after platinum based therapy and at least one other line of therapy |
| Olaparib | AstraZeneca | 2017 - recurrent epithelial ovarian, fallopian tube, or pirmary peritoneal cancer, who are in CR or PR to platinum-based therapy  2018 – metastatic HER2 negative breast cancer with BRCA-mutation, with prior chemotherapy  2018 - Maintenance treatment of recurrent ovarian, fallopian tube, or primary peritoneal cancer after response to first line platinum based therapy  2019 – 1^st^ line maintenance treatment of germline BRCA-mutated metastatic pancreatic cancer |
| Osimertinib | AstraZeneca | 2015 – metastatic EGFR positive NSCLC, second line  2017 – metastatic EGFR T790M mutation after EGFR TKI therapy  2018 – 1^st^ line metastatic NSCLC with EGFR exon 19 deletion or exon 21 L858R mutation |
| Palbociclib | Pfizer | 2015 – combined with aromatase inhibitor for advanced breast cancer  2016 – metastatic hormone-receptor positive breast cancer with disease progression after endocrine therapy  2017 - metastatic hormone-receptor positive breast cancer as first line with endocrine therapy |
| Pembrolizumab | Merck | 2015 – metastatic NSCLC 2^nd^ PD-L1 positive, post platinum chemotherapy  2015 – metastatic melanoma, 2^nd^ line therapy  2016 – recurrent/metastatic head and neck squamous cell carcinoma after platinum-therapy  2016 – 1^st^ line therapy for metastatic NSCLC, PD-L1 ≥ 50%, no EGFR/ALK mutation  2016 – 2^nd^ line therapy for metastatic NSCLC, PD-L1 ≥ 1%, no EGFR/ALK mutation, with disease progression on platinum-based therapy  2017 – 1^st^ line therapy for non-squamous NSCLC in combination with carboplatin & pemetrexed  2017 – metastatic urothelial cancer after platinum based therapy  2017 – adult metastatic MSI-high or dMMR cancers without alternative systemic options  2017 – metastatic gastric or gastro-esophageal junction cancer with PD-L1 expression  2018 – Metastatic cervical cancer whose tumours express PD-L1 after chemotherapy  2018 – 1^st^ line metastatic non-squamous NSCLC (no ALK or EGFR mutation) in combination with platinum and pemetrexed  2018 – 1^st^ line for metastatic squamous NSCLC in combination with carboplatin and taxane  2018 – metastatic HCC previously treated with sorafenib  2018 – 1^st^ line metastatic Merkel cell carcinoma  2019 – Adjuvant lymph node positive melanoma following surgery  2019 - Stage 3 NSCLC first line who are not candidates for surgical resection or definitive chemoradation if PD-L1 ≥1%  2019 – 1^st^ line metastatic head and neck squamous cell cancer with platinum based therapy for PD-L1 ≥1%  2019 – metastatic SCLC after platinum  2019 – metastatic squamous cell esophageal carcinoma with PD-L1 ≥ 1% after progression on one or more prior lines of systemic therapy |
| Pertuzumab | Roche | 2017 – Adjuvant treatment for HER-2 positive breast cancer, in combination with trastuzumab |
| Pexidartinib | Daiichi Sankyo | 2019 – adults with symptomatic tenosynovial giant cell tumor associated with severe morbidity or functional limitations that are not amenable to improvement with surgery |
| Ramucirumab | Eli Lilly | 2015 – 2^nd^ line, combination with FOLFIRI  2019 – 2^nd^ line for HCC after sorafenib with AFP > 400 |
| Regorafenib | Bayer | 2017 – metastatic HCC previously treated with sorafenib |
| Ribociclib | Novartis and Astex | 2017 – 1^st^ line with endocrine therapy for post-menopausal metastatic hormone-receptor positive, HER2 negative, breast cancer  2018 - 1^st^ line with endocrine therapy for pre/peri-menopausal metastatic hormone-receptor positive, HER2 negative, breast cancer |
| Rucaparib | Clovis / Pharmaand GmbH | 2016 – advanced ovarian cancer with BRCA mutations after two or more chemotherapies  2018 - maintenance treatment of recurrent ovarian, fallopian tube, or primary peritoneal cancer |
| Sonidegib | Sun Pharmaceutical | 2015 – recurrent/locally Advanced Basal Cell Carcinoma |
| Sunitinib | Pfizer | 2017 – high risk recurrent RCC, adjuvant following nephrectomy |
| Talazoparib | Pfizer | 2018 – metastatic HER-2/negative breast cancer with BRCA mutations |
| Trabectedin | Janssen Biotech | 2015 - metastatic liposarcoma or leiomyosarcoma post anthracycline |
| Trastuzumab Deruxtecan | Daiichi Sankyo | 2019 – metastatic HER2-positive breast cancer with two or more prior lines of anti-HER2-based regimens in the metastatic setting |
| Trastuzumab Emtansine (T-DM1) | Genetech / Lonza | 2019 – adjuvant therapy for HER2 positive breast cancer with residual disease after neoadjuvant taxane and trastuzumab |
| Trifluridine/tipiracil (TAS-102) | Taiho Pharmaceutical | 2015 – metastatic CRC, RAS wt, after several lines of therapy  2019 – metastatic gastric or gastro-esophageal junction cancer after two prior lines of chemotherapy |

Note 1: In table above, an approval in the metastatic setting typically includes ‘locally advanced unresectable’

CRC – colorectal carcinoma

HCC – hepatocellular carcinoma

NSCLC – non-small cell lung cancer

RCC – renal cell carcinoma

SCLC – small cell lung cancer

TKI – tyrosine kinase inhibitor
