# Supplementary material for: Magnitude of effect and sample size justification in trials supporting anti-cancer drug approval by the US Food and Drug Administration
Source: Sci Rep. 2024 Jan 3;14:459. doi: 10.1038/s41598-023-50694-0 (PMC10764749; doi:10.1038/s41598-023-50694-0)
Supplement: Supplementary file 2 — Supplementary Information. [file 41598_2023_50694_MOESM2_ESM.docx]

Supplementary Table 1: Supplementary methods and assumptions

| - If the alpha was 1-sided, it was adjusted to 2-sided by multiplying by 2. |
| --- |
| - Supplementary files of published articles were reviewed only if the information was not reported clearly in the manuscript. In the event of a discrepancy between these information sources, data were extracted preferentially from the manuscript. |
| - If only the month of accrual time was provided, we assumed that accrual began on the first of the month and ended on the last day of the given month. |
| - If the median number of months of the outcome of interest for the control arm was not available, it was calculated using the following formula: *t* log_e_(1/2)/log_e_(*p*) where *p* is the probability that a control subject survives until time *t*. For these cases the proportion of patients with the outcome of interest at time *t* was extracted from the trial report. |
| - For any overall survival end-point, if the median number of months was not provided (as in previous bullet point) *and* more than one estimate of survival was reported (i.e. at different time points), we calculated the median time for both time points and used the mean value. |

Supplementary Tables 3: Sensitivity Analyses performed to define the proportion of over-sampled end-points

3A: Power Equivalent if absolute difference ± 2.5%, HR equivalent if absolute difference ± 1%

|  | **{P_obs_ > (P_exp_ +2.5%)}**  **(n=69)**  **(2)** | **Well Powered**  **{P_obs_ = (P_exp_ +/-2.5%)}**  **(n=3)**  **(1)** | **Under-powered**  **{P_obs_ < (P_exp_ – 2.5%)}**  **(n=19)**  **(3)** |
| --- | --- | --- | --- |
| *HR_obs_* better magnitude of effect than *HR_exp_* (n=55) (2) | 53 | 1 | 1 |
| *HR_obs_* similar magnitude of effect as *HR_exp_* (n=11) (1) | 9 | 0 | 2 |
| *HR_obs_* worse magnitude of effect than *HR_exp_* (n=25) (3) | 7 | 2 | 16 |

Over-sampled: 18 / 91 (20%)

3B: Power Equivalent if absolute difference ± 2.5%, HR equivalent if absolute difference ± 2.5%

|  | **{P_obs_ > (P_exp_ +2.5%)}**  **(n=69)** | **Well Powered**  **{P_obs_ = (P_exp_ +/-2.5%)}**  **(n=3)** | **Under-powered**  **{P_obs_ < (P_exp_ – 2.5%)}**  **(n=19)** |
| --- | --- | --- | --- |
| *HR_obs_* better magnitude of effect than *HR_exp_* (n=54) | 52 | 1 | 1 |
| *HR_obs_* similar magnitude of effect as *HR_exp_* (n=15) | 12 | 0 | 3 |
| *HR_obs_* worse magnitude of effect than *HR_exp_* (n=22) | 5 | 2 | 15 |

Over-sampled: 17 / 91 (19%)

3C: Power Equivalent if absolute difference ± 2.5%, HR equivalent if absolute difference ± 5%

|  | **{P_obs_ > (P_exp_ +2.5%)}**  **(n=69)**  **(2)** | **Well Powered**  **{P_obs_ = (P_exp_ +/-2.5%)}**  **(n=3)**  **(1)** | **Under-powered**  **{P_obs_ < (P_exp_ – 2.5%)}**  **(n=19)**  **(3)** |
| --- | --- | --- | --- |
| *HR_obs_* better magnitude of effect than *HR_exp_* (n=46) (2) | 45 | 0 | 1 |
| *HR_obs_* similar magnitude of effect as *HR_exp_* (n=32) (1) | 22 | 1 | 9 |
| *HR_obs_* worse magnitude of effect than *HR_exp_* (n=13) (3) | 2 | 2 | 9 |

Over-sampled: 26 / 91 (29%)

3D: Power Equivalent if absolute difference ± 5%, HR equivalent if absolute difference ± 1%

|  | **{P_obs_ > (P_exp_ +5%)}**  **(n=56)**  **(2)** | **Well Powered**  **{P_obs_ = (P_exp_ +/-5%)}**  **(n=18)**  **(1)** | **Under-powered**  **{P_obs_ < (P_exp_ – 5%)}**  **(n=17)**  **(3)** |
| --- | --- | --- | --- |
| *HR_obs_* better magnitude of effect than *HR_exp_* (n=55) (2) | 47 | 7 | 1 |
| *HR_obs_* similar magnitude of effect as *HR_exp_* (n=11) (1) | 7 | 4 | 7 |
| *HR_obs_* worse magnitude of effect than *HR_exp_* (n=25) (3) | 3 | 7 | 15 |

Over-sampled: 17 / 91 (19%)

3E: Power Equivalent if absolute difference ± 5%, HR equivalent if absolute difference ± 2.5%

|  | **{P_obs_ > (P_exp_ +5%)}**  **(n=56)**  **(2)** | **Well Powered**  **{P_obs_ = (P_exp_ +/-5%)}**  **(n=18)**  **(1)** | **Under-powered**  **{P_obs_ < (P_exp_ – 5%)}**  **(n=17)**  **(3)** |
| --- | --- | --- | --- |
| *HR_obs_* better magnitude of effect than *HR_exp_* (n=15) (2) | 46 | 7 | 1 |
| *HR_obs_* similar magnitude of effect as *HR_exp_* (n=54) (1) | 8 | 6 | 1 |
| *HR_obs_* worse magnitude of effect than *HR_exp_* (n=22) (3) | 2 | 5 | 15 |

Over-sampled: 15 / 91 (16%)

3F: Power Equivalent if absolute difference ± 5%, HR equivalent if absolute difference ± 5%

|  | **{P_obs_ > (P_exp_ +5%)}**  **(n=56)**  **(2)** | **Well Powered**  **{P_obs_ = (P_exp_ +/-5%)}**  **(n=18)**  **(1)** | **Under-powered**  **{P_obs_ < (P_exp_ – 5%)}**  **(n=17)**  **(3)** |
| --- | --- | --- | --- |
| *HR_obs_* better magnitude of effect than *HR_exp_* (n=46) (2) | 42 | 3 | 1 |
| *HR_obs_* similar magnitude of effect as *HR_exp_* (n=32) (1) | 13 | 12 | 7 |
| *HR_obs_* worse magnitude of effect than *HR_exp_* (n=13) (3) | 1 | 3 | 9 |

Over-sampled: 17 / 91 (19%)

Supplemental Table 4A: Sensitivity Analysis excluding all studies where follow-up time after end of accrual was 0 (F=0) as database lock and end of accrual occurred simultaneously (n=85)

|  | **{P_obs_ > (P_exp_ +2.5%)}**  **(n=65)** | **Well Powered**  **(n=3)** | **Under-powered**  **{P_obs_ < (P_exp_ - 2.5%)}**  **(n=17)** |
| --- | --- | --- | --- |
| *HR_obs_* better magnitude of effect than *HR_exp_* (n=51) | 49 | 1 | 1 |
| *HR_obs_* similar magnitude of effect as *HR_exp_* (n=13) | 11 | 0 | 2 |
| *HR_obs_* worse magnitude of effect than *HR_exp_* (n=21) | 5 | 2 | 14 |

Over-sampled: 18 / 85 (21%)

Supplemental Table 4B: Sensitivity Analysis excluding all studies where follow-up time after end of accrual was estimated (F estimated, could have been F=0 or F>0) (n=85)

|  | **{P_obs_ > (P_exp_ +2.5%)}**  **(n=64)** | **Well Powered**  **(n=3)** | **Under-powered**  **{P_obs_ < (P_exp_ - 2.5%)}**  **(n=18)** |
| --- | --- | --- | --- |
| *HR_obs_* better magnitude of effect than *HR_exp_* (n=50) | 48 | 1 | 1 |
| *HR_obs_* similar magnitude of effect as *HR_exp_* (n=14) | 11 | 0 | 3 |
| *HR_obs_* worse magnitude of effect than *HR_exp_* (n=21) | 5 | 2 | 14 |

Over-sampled: 18 / 85 (21%)

Supplemental Table 4C: Sensitivity Analysis excluding all studies where follow-up time after end of accrual was derived *OR* was zero (F estimated or F=0) (n=80)

|  | **{P_obs_ > (P_exp_ +2.5%)}**  **(n=61)** | **Well Powered**  **(n=3)** | **Under-powered**  **{P_obs_ < (P_exp_ - 2.5%)}**  **(n=16)** |
| --- | --- | --- | --- |
| *HR_obs_* better magnitude of effect than *HR_exp_* (n=48) | 46 | 1 | 1 |
| *HR_obs_* similar magnitude of effect as *HR_exp_* (n=12) | 10 | 0 | 2 |
| *HR_obs_* worse magnitude of effect than *HR_exp_* (n=20) | 5 | 2 | 13 |

Over-sampled: 17 / 80 (21%)

Supplemental Table 4D: Sensitivity Analysis excluding studies were median outcome of interest was calculated (n=82)^*^

|  | **{P_obs_ > (P_exp_ +2.5%)}**  **(n=62)** | **Well Powered**  **(n=3)** | **Under-powered**  **{P_obs_ < (P_exp_ - 2.5%)}**  **(n=17)** |
| --- | --- | --- | --- |
| *HR_obs_* better magnitude of effect than *HR_exp_* (n=48) | 46 | 1 | 1 |
| *HR_obs_* similar magnitude of effect as *HR_exp_* (n=13) | 11 | 0 | 2 |
| *HR_obs_* worse magnitude of effect than *HR_exp_* (n=20) | 5 | 2 | 14 |

^*^ 10 studies had a median that was derived as it was not reported, however 1 was also missing HR_exp_ and therefore excluded from this analysis at an earlier stage.

Over-sampled: 18 / 82 (22%)

Supplemental Table 5: Sensitivity Analysis looking at only 1 endpoint per trial (hierarchy: primary endpoint over secondary, and OS over surrogates) (n=76)

|  | **{P_obs_ > (P_exp_ +2.5%)}**  **(n=59)** | **Well Powered**  **(n=0)** | **Under-powered**  **{P_obs_ < (P_exp_ - 2.5%)}**  **(n=17)** |
| --- | --- | --- | --- |
| *HR_obs_* better magnitude of effect than *HR_exp_* (n=46) | 45 | 0 | 1 |
| *HR_obs_* similar magnitude of effect as *HR_exp_* (n=14) | 11 | 0 | 3 |
| *HR_obs_* worse magnitude of effect than *HR_exp_* (n=16) | 3 | 0 | 13 |

Over-sampled: 14 / 76 (18%)

Supplemental Table 6A: Sensitivity regression analysis of Trial characteristics of Over- and Under-sampled Endpoints, including only 1 endpoint per study to avoid co-linearity (n=76)

|  | Over-sampled Studies (n=14) | Not Over-sampled  (n=62) | OR | P |
| --- | --- | --- | --- | --- |
| Expected Power (%) | 87.5+/-4.6 | 86.6+/-8.2 | 1.02 | 0.69 |
| Alpha (%) | 5.4 +/- 5.6 | 5.6 +/- 6.0 | 0.99 (0.897-1.10) | 0.92 |
| Sample Size – exp arm | 328.3 +/- 339.1 | 390.3 +/- 340.4 | 0.999 (0.997-1.001) | 0.52 |
| m1 control (months) | 21.2 +/- 48.6 | 19.5 +/- 45.8 | 1.00 (0.99-1.01) | 0.90 |
| Study Year  2015, n(%)  2016, n(%)  2017, n(%)  2018, n(%)  2019, n(%) | 5 (35.7%)  1 (7.1%)  4 (28.6%)  0  4 (28.6%) | 12 (19.3%)  7 (11.3%)  12 (19.3%)  19 (30.3%)  12 (19.3%) | 0.82 (0.55-1.23) | 0.34^*^ |
| Type of Therapy^*^  Other, n(%)  Targeted Therapy, n(%)  Immunotherapy, n(%) | 2 (14.3%)  5 (35.7%)  7 (50.0%) | 21 (33.9%)  23 (37.1)  18 (29.0%) | 1  2.28 (0.49-13.05)  4.08 (0.75-22.19) | 0.35  0.10 |
| Type of Endpoint  Other, n(%)  OS, n(%) | 39 (62.9%)  23 (37.1%) | 7 (50.0%)  7 (50.0%) | 1  1.69 (0.53 – 5.56) | 0.38 |
| Disease Site  Breast, n(%)  Lung, n(%)  Melanoma, n(%)  Other, n(%) | 2 (14.3%)  4 (28.6%)  3 (21.4%)  5 (35.7%) | 12 (19.3%)  14 (22.6%)  7 (11.3%)  29 (46.8%) | 1  1.7 (0.26-11.06)  2.57 (0.34-19.33)  1.03 (0.17-6.09) | 0.57  0.36  0.97 |
| Blinding  Double Blind, n(%)  Open Label, n(%) | 6 (42.9%)  8 (57.1%) | 37 (59.7%)  25 (40.3%) | 1  1.96 (0.61-6.25) | 0.26 |
| Loss to Follow-up or Withdrawal (mean +/- SD) | 19.9 +/- 16.0 | (n=61)  25.3 +/- 35.8 | 0.99 (0.97 – 1.02) | 0.58 |

^*^P for trend

Supplemental Table 6B: Sensitivity regression analysis of Over- and Under-sampled Endpoints using Power Equivalent if absolute difference ± 5%, HR equivalent if absolute difference ± 2.5% (Oversampled trials from Supplementary figure 2E: 16% of end-points over-sampled)

|  | Over-sampled Studies (n=15) | Not Over-sampled  (n=76) | OR | P |
| --- | --- | --- | --- | --- |
| Expected Power (%) | 88.1+/-6.04 | 87.1+/-7.9 | 1.02 (0.93-1.11) | 0.64 |
| Alpha (%) | 4.75+/-5.66 | 5.00+/-5.60 | 0.98 (0.88-1.09) | 0.73 |
| Sample Size – exp arm | 272+/-164 | 397+/-317 | 0.99 (0.99-1.00) | 0.63 |
| m1 control (months) | 8.38 +/-4.83 | 20.1+/-46.0 | 1.00 (0.98-1.01) | 0.98 |
| Study Year  2015, n(%)  2016, n(%)  2017, n(%)  2018, n(%)  2019, n(%) | 5 (33.3%)  1 (6.7%)  3 (20.0%)  0  6 (40.0%) | 14 (18.4%)  9 (11.8%)  14 (18.4%)  24 (31.6%)  15 (19.7%) | 0.92 (0.63-1.35) | 0.70 |
| Type of Therapy^*^  Other, n(%)  Targeted Therapy, n(%)  Immunotherapy, n(%) | 2 (13.3%)  4 (26.7%)  9 (60.0%) | 24 (31.6%)  26 (34.2%)  26 (34.2%) | 1  1.85 (0.31-11.01)  4.15 (0.81-21.19) | 0.50  0.87 |
| Type of Endpoint  Other, n(%)  OS, n(%) | 8 (53.3%)  7 (46.7%) | 50 (65.8%)  26 (34.2%) | 1  1.69 (0.55-5.26) | 0.36 |
| Disease Site  Breast, n(%)  Lung, n(%)  Melanoma, n(%)  Other, n(%) | 1 (6.7%)  4 (26.7%)  4 (26.7%)  6 (40.0%) | 14 (18.4%)  20 (26.3%)  8 (10.5%)  34 (44.7%) | 1  2.8 (0.28-27.8)  7.0 (0.66-73.9)  2.47 (0.27-22.4) | 0.37  0.11  0.42 |
| Blinding  Double Blind, n(%)  Open Label, n(%) | 6 (40%)  9 (60%) | 44 (57.9%)  32 (42.1%) | 1  2.03 (0.67-6.67) | 0.21 |
| Loss to Follow-up or Withdrawal (mean +/- SD) | 24.5+/-17.7 | 24.8+/-33.1 | 0.99 (0.98-1.02) | 0.98 |

^*^P for trend

Supplemental Table 6C: Sensitivity regression analysis of Over- and Under-sampled Endpoints using Power Equivalent if absolute difference ± 2.5%, HR equivalent if absolute difference ± 5%

(Oversampled trials from Supplementary figure 2C: 29% of end-points over-sampled)

|  | Over-sampled Studies (n=26) | Not Over-sampled  (n=65) | OR | P |
| --- | --- | --- | --- | --- |
| Expected Power (%) | 87.6+/-11.0 | 87.1+/-5.9 | 1.01 (0.95-1.07) | 0.82 |
| Alpha (%) | 4.38+/-4.4 | 5.2+/-6.0 | 0.97 (0.87-1.07) | 0.54 |
| Sample Size – exp arm | 318+/-263 | 400+/-312 | 0.99 (0.99-1.00) | 0.63 |
| m1 control (months) | 15.8+/-35.8 | 19.1+/-44.8 | 0.99 (0.98-1.01) | 0.74 |
| Study Year  2015, n(%)  2016, n(%)  2017, n(%)  2018, n(%)  2019, n(%) | 9 (34.6%)  3 (11.5%)  5 (19.2%)  1 (3.8%)  8 (30.8%) | 10 (15.4%)  7 (10.8%)  12 (18.5%)  23 (35.4%)  13 (20.0%) | 0.79 (0.58-1.08) | 0.15^*^ |
| Type of Therapy^*^  Other, n(%)  Targeted Therapy, n(%)  Immunotherapy, n(%) | 3 (11.5%)  8 (30.1%)  15 (57.7%) | 23 (35.4%)  22 (33.8%)  20 (30.8%) | 1  2.79 (0.65-11.89)  5.75 (1.45-22.78) | 0.17  0.013 |
| Type of Endpoint  Other, n(%)  OS, n(%) | 15 (57/7%)  11 (42.3%) | 43 (66.2%)  22 (33.8%) | 1  1.43 (0.56- 3.7) | 0.45 |
| Disease Site  Breast, n(%)  Lung, n(%)  Melanoma, n(%)  Other, n(%) | 2 (7.7%)  8 (30.8%)  7 (26.9%)  9 (34.6%) | 13 (20.0%)  16 (24.6%)  5 (7.7%)  31 (47.7%) | 1  3.25 (0.58-18.03)  9.1 (1.38-59.62)  1.89 (0.35-9.95) | 0.18  0.02  0.45 |
| Blinding  Double Blind, n(%)  Open Label, n(%) | 9 (34.6%)  17 (65.4%) | 41 (63.1%)  24 (36.9%) | 1  3.22 (1.25-8.33) | 0.016 |
| Loss to Follow-up or Withdrawal (mean +/- SD) | 20.5+/-17.2 | 26.5+/-35.1 | 0.99 (0.97-1.01) | 0.41 |

^*^P for trend

Supplemental Figure 1: Histogram demonstrating degree of difference in power (ΔP_O-E_ ) of all endpoints (n=94)

**
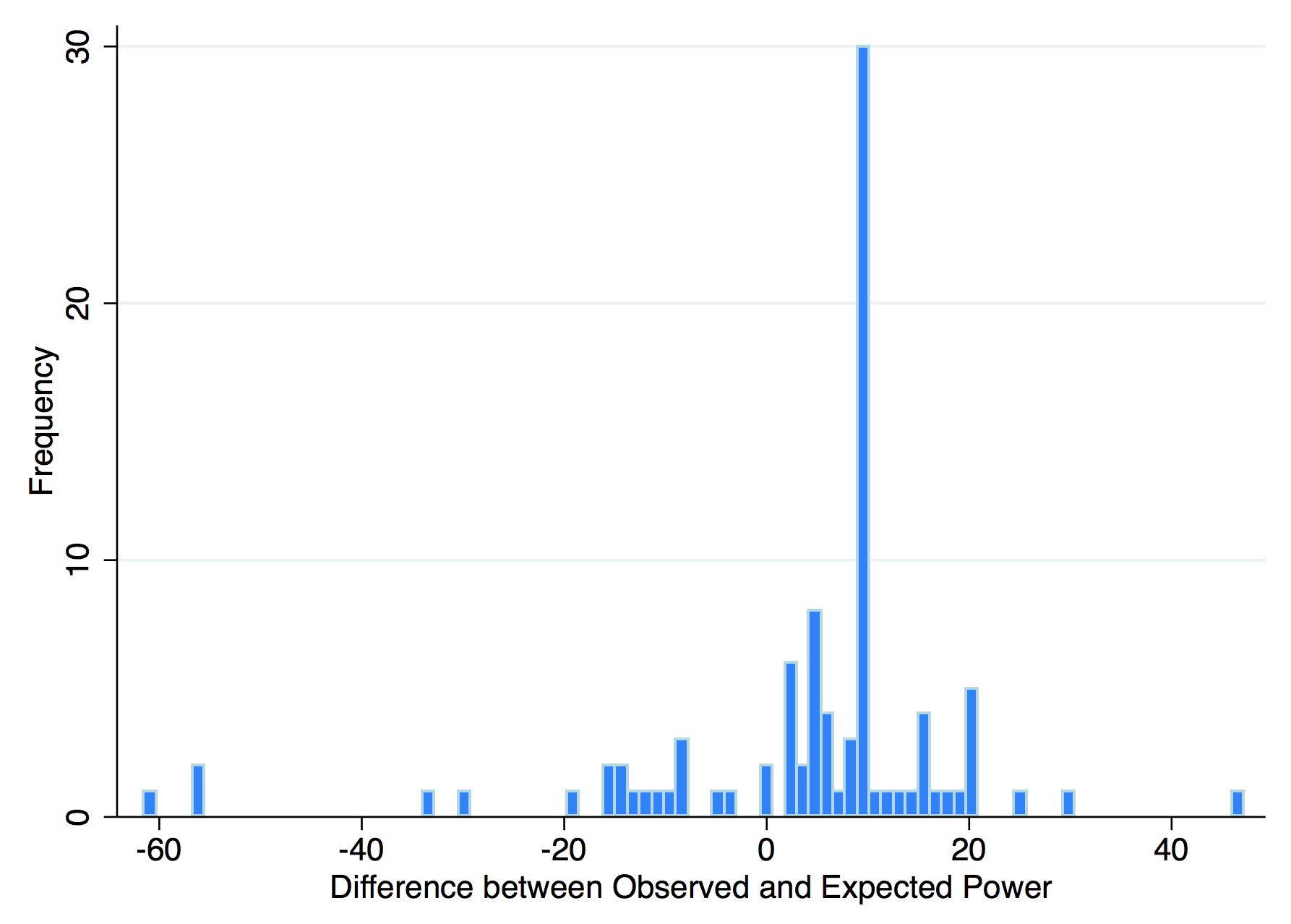
**

[P_obs_-P_exp_)*100] > 2.5 (n=72)

Studies are underpowered if [P_obs_-P_exp_)*100] < -2.5 (n=3)

Studies are similarly powered if [P_obs_-P_exp_)*100] is between -2.5 and +2.5 (n=19)

Supplemental Figure 2: Histogram demonstrating degree of difference in HR of all available endpoints (n=91)

**
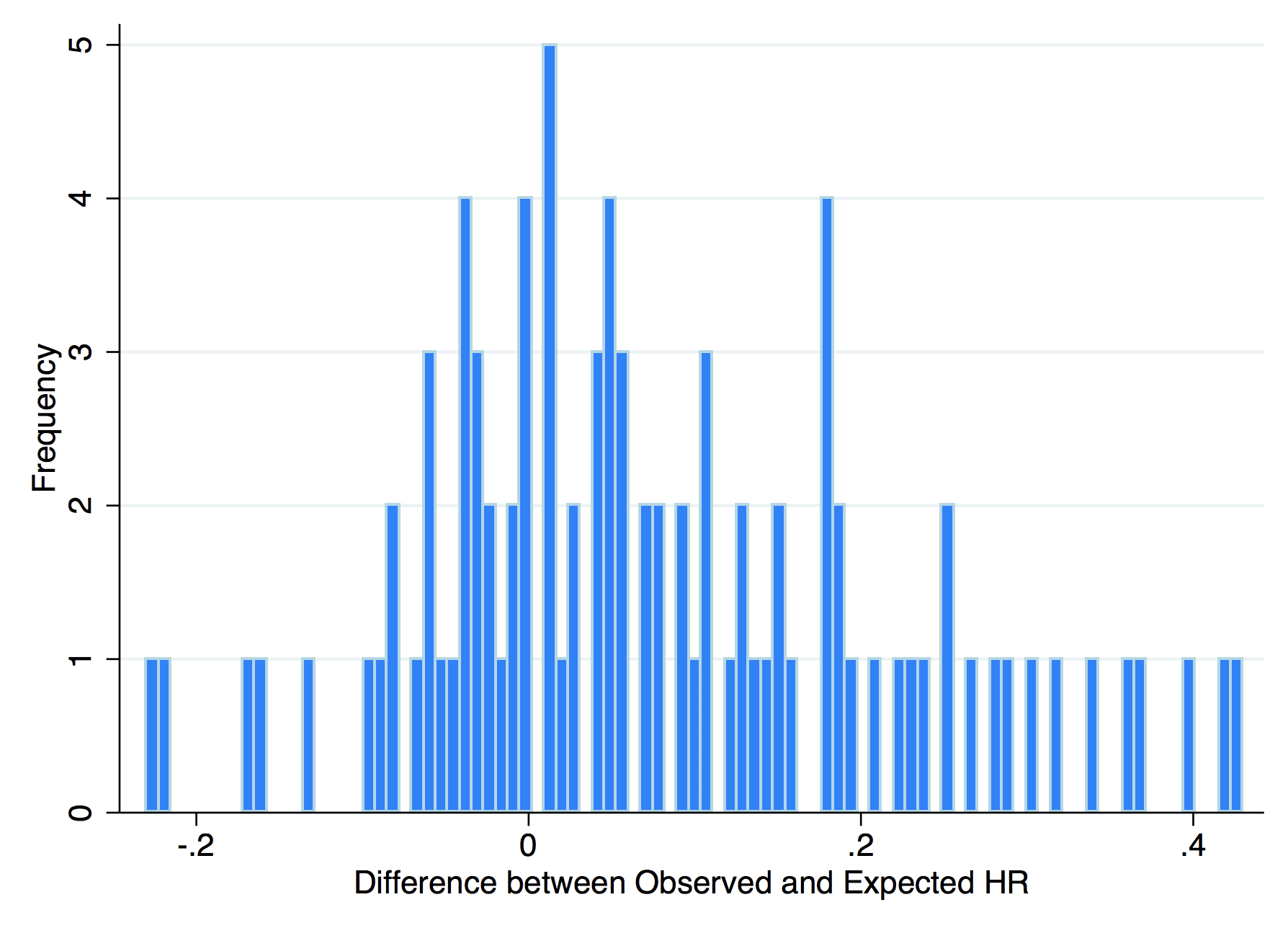
**
